# Examining the impact of acetylene on N-fixation and the active sediment microbial community

**DOI:** 10.3389/fmicb.2015.00418

**Published:** 2015-05-12

**Authors:** Robinson W. Fulweiler, Elise M. Heiss, Mary Kate Rogener, Silvia E. Newell, Gary R. LeCleir, Sarah M. Kortebein, Steven W. Wilhelm

**Affiliations:** ^1^Department of Earth and Environment, Boston UniversityBoston, MA, USA; ^2^Department of Biology, Boston UniversityBoston, MA, USA; ^3^Department of Chemistry and Physics, King’s CollegeWilkes-Barre, PA, USA; ^4^Department of Marine Sciences, University of GeorgiaAthens, GA, USA; ^5^Department of Earth and Environmental Sciences, Wright State UniversityDayton, OH, USA; ^6^Department of Microbiology, University of TennesseeKnoxville, TN, USA

**Keywords:** heterotrophic nitrogen fixation, acetylene reduction assay, sediments, sulfate-reducing bacteria, high throughput sequencing

## Abstract

Here we examined the impact of a commonly employed method used to measure nitrogen fixation, the acetylene reduction assay (ARA), on a marine sediment community. Historically, the ARA technique has been broadly employed for its ease of use, in spite of numerous known artifacts. To gauge the severity of these effects in a natural environment, we employed high-throughput 16S rRNA gene sequencing to detect differences in acetylene-treated sediments vs. non-treated control sediments after a 7 h incubation. Within this short time period, significant differences were seen across all activity of microbes identified in the sediment, implying that the changes induced by acetylene occur quickly. The results have important implications for our understanding of marine nitrogen budgets. Moreover, because the ARA technique has been widely used in terrestrial and freshwater habitats, these results may be applicable to other ecosystems.

## Introduction

Every sampling effort and each experimental design impacts the environment and the processes we wish to measure. Quantifying the nature and extent of that effect is a necessary step in our broader understanding of microbial processes and for the interpretation of the data we collect. High-throughput 16S rRNA gene-based bacterial community surveys provide a powerful mechanism for us to examine the impact of commonly employed geochemical methods on the microbial population.

Of particular interest to many aquatic biogeochemists is the microbially-mediated process of nitrogen fixation (N-fixation), which converts inert dinitrogen (N_2_) gas to biologically reactive nitrogen (N). While Earth’s atmosphere is comprised primarily of N_2_ gas, most organisms cannot tap into this reservoir. Thus, N-fixation provides critical links between biological organisms, this unreactive N pool, and N concentrations in the environment. Indeed, the ability to fix N_2_ provides a significant advantage to organisms with this capability. Once N is fixed, various processes transform reactive N until it is returned to the atmosphere through denitrification. Because N is an essential and often limiting nutrient for primary productivity, its availability, at least in part, constrains global primary productivity (Tyrrell, 1999; Elser et al., 2007).

Direct measurements of N-fixation rates from decreases in N_2_ concentration are difficult, as they require detection of small changes in a large reservoir of N_2_ gas. Equally challenging is the measurement of *in situ* total N increases to quantify N fixation, because this method also requires detection of a small amount of N against a much larger background of environmental organic nitrogen (Seitzinger and Garber, 1987). In recent years, our ability to measure such small changes has increased substantially, but they are time consuming and expensive. As such, for the last five decades we have used the methodologically more simple acetylene reduction assay (ARA) to measure N-fixation (Dilworth, 1966; Hardy et al., 1968; Stewart et al., 1968). The ARA relies on the flexibility of nitrogenase, the enzyme responsible for N_2_ reduction, as it can reduce acetylene (C_2_H_2_) to ethylene (C_2_H_4_), which can then be directly quantified. Theoretically, every three moles of ethylene produced corresponds to the reduction of 1 mole of N_2_ to ammonia.

However, the ARA is known to have numerous experimental artifacts. Some of these are newly discovered and related to the addition of acetylene and subsequent incubation of the sample (Mohr et al., 2010; Wilson et al., 2012). Other factors compromising this technique have been known for much longer and are related to shifts in the activity and ability of the microbial community to process nutrients and carbon in the presence of acetylene. For example, acetylene irreversibly inhibits methanogenesis in cultures and sediments (Oremland and Taylor, 1975). Acetylene also reversibly blocks aerobic nitrification (Hynes and Knowles, 1982) and nitrous oxide reduction in anaerobic denitrifiers (Balderston et al., 1976). Almost thirty years ago, acetylene was shown to partially or completely inhibit carbon dioxide production and growth of two sulfate reducing bacteria, *Desulfovibrio desulfuricans* and *Desulfovibrio gigas* although another species, *Desulfotomaculum ruminis*, was not effected at all (Payne and Grant, 1982). This is particularly relevant for marine ecosystems where sulfur- and sulfate-reducing bacteria are often significant contributors to the N-fixing community (Nielsen et al., 2001; Bertics et al., 2013; Brown and Jenkins, 2014).

To examine the broader applicability of the foundational efforts of Payne and Grant (1982), we used sediments from a temperate New England estuary to investigate the influence of the ARA on the total active microbial community of marine sediment. We directly measured net N_2_ fluxes and then measured N-fixation using ARA. Following the ARA, we conducted a 16S rRNA bacterial gene community survey to examine the change in active community activity after exposure to acetylene. The results are provided in the context of how the ARA technique alters processes within the community it is intended to unobtrusively query.

## Materials and Methods

### Study Site and Sample Collection

Samples were collected in July 2011 from a station in Narragansett Bay, RI, USA (41°35.3′, 071°22.3′) where the water column depth was 8 m. The *in situ* temperature was 17°C and the salinity was 31.3 psu. Intact triplicate sediment cores were hand collected by SCUBA divers with the vertical architecture of the sediment maintained during core collection and handling (Fulweiler and Heiss, 2014). Capped cores were transported with *in situ* water headspace in coolers filled with site water to an environmental chamber set to ambient temperature at the Graduate School of Oceanography at the University of Rhode Island. The sediment cores were placed in a 17°C water bath in the dark with air gently bubbling through the surface water until the incubation began (∼8 h).

### Net N_2_ Measurements

We first completed an N_2_/Ar incubation to measure the net flux of N_2_ across the sediment-water interface. These methods have been documented previously (Fulweiler and Nixon, 2009, 2012). Briefly, before the incubation we carefully replaced the overlying water with filtered (0.2-μm) site water, the cores were sealed with a gas tight lid (no air headspace), gently stirred (∼40 rpm), and replicate samples taken for N_2_/Ar analysis at five points over the course of an incubation. Each sample was preserved with 20 μl of saturated ZnCl solution. All incubations took place in the dark and lasted ∼ 11 h until we observed an overlying water oxygen concentration drop of at least 2 mg L^-1^ (62.5 μM), but at no point did the cores approach hypoxia. Dissolved N_2_ and Ar gas concentrations were analyzed on a quadrupole membrane inlet mass spectrometer with a precision of ± 0.03% (Sisler and ZoBell, 1951; Heip, 1995; Kana et al., 1998). N_2_ change for each of the triplicate cores was determined from a five-point linear regression. Rates were then prorated for the volume of water overlying the core and the sediment area of the core. The rates calculated from the N_2_/Ar technique actually present a measure of net N_2_ flux (gross denitrification – gross N fixation).

### ARA Measurements

After net N_2_ measurements were made, we quantified the reduction of acetylene (C_2_H_2_) to ethylene (C_2_H_4_) as a proxy for N-fixation (Hardy et al., 1968). Duplicate sub-cores (∼2.5 cm i.d.) were collected from each core and sectioned into 0–2m and 2–4 cm increments, halved, and placed in 40 mL glass vials. For each depth, each core duplicate sample halves were separated into the following treatments: acetylene plus filtered seawater, acetylene plus 40 mM sodium molybdate (Na_2_MoO_4_), seawater alone, and 40 mM Na_2_MoO_4_ alone. Molybdate is an established inhibitor of sulfate-reducing bacteria and used as an indicator of N-fixation by sulfate reducing bacteria (Postgate, 1949; Oremland and Taylor, 1978). Samples were placed in sealed vials, the air headspace was evacuated, and replaced with argon gas after which 10 mL of C_2_H_2_ was injected into the vials (Hamilton et al., 2011; Cole and McGlathery, 2012). The ARA incubation lasted 7 h. Gas samples were run on a Shimadzu gas chromatograph equipped with a flame ionization detector using a Porapak N column, mesh size 80/100 at the Graduate School of Oceanography at the University of Rhode Island. The halved sub-core samples were then extrapolated to area units and rates of sediment acetylene reduction were converted to N-fixed using the common 3:1 molar conversion (Seitzinger and Garber, 1987). All samples were then frozen at -80°C and shipped overnight to the University of Tennessee for molecular analysis.

### Molecular Analysis

RNA was extracted from all 24 sediment samples using the MoBio PowerSoil^TM^ RNA isolation kit (MoBio, Carlsbad, CA, USA) according to manufacturer’s protocols and checked for contaminating DNA by PCR (below). cDNA was produced from extracted RNA using 16S rRNA gene-specific primers (9F and 1522R, *E. coli* numbering) using the Invitrogen Superscript III First-Strand Synthesis SuperMix kit according the manufacturer’s protocols. We PCR amplified bacterial 16S rRNA using primers targeting bases 338–926 of the 16S rRNA gene (*E. coli* numbering), which contains the V3–V5 region, with the following PCR protocol: 95°C for 5 min, followed by 30 rounds of (95°C for 30 s, 55°C for 30 s, 72°C for 30 s) and then a final extension step at 72°C for 10 min. Product amplification was verified on a 1% agarose gel stained with ethidium bromide and viewed on a UV transilluminator. Individual samples were processed to remove unincorporated primers and nucleotides using the Qiaquick PCR cleanup kit (Qiagen, Valencia, CA, USA). Amplicon concentrations were determined using a NanoDrop ND-1000 spectrophotometer (Thermo Scientific, Wilmington, DE, USA).

Individual sample amplicons were barcoded (six additional PCR cycles : 95°C for 30 s, 55°C for 30 s, 72°C for 30 s) with primers that contained a unique 8-bp barcode attached to the 454 fusion primers (LeCleir et al., 2014; Wilhelm et al., 2014). The barcoding primers were designed for unidirectional sequencing on a 454 GSFLX sequencer (454 Life Sciences, Branford, CT, USA). This strategy required the use of the Lib-L kit (see Roche application brief 001-2009). We opted for unidirectional sequencing as our PCR product was larger than the average read length of the available 454 Titanium sequencing chemistry. This approach ensured sequences would overlap for the longest length possible. All barcoding reactions were prepared to have 0.5 ng/μl of amplicon DNA per reaction. After the barcoding reaction, we again verified our amplicons on an agarose gel before pooling all barcoded amplicons. Barcoded amplicons were processed to remove unincorporated primers and nucleotides using a single Qiagen Qiaquick column. Sequencing was completed at the University of Tennessee/Oak Ridge National Laboratory Joint institute of Biological Sciences. Sequence information has been deposited in the NCBI short-read archive in bioproject PRJNA271790.

We used the Mothur software package (version 1.27.0; Schloss et al., 2009) to process our sequences for sufficient length and quality. We processed our sequences similar to the Schloss SOP^1^ with some modifications of the shhh.flows command; we changed the number of flows value in the shhh.flows command to 360–720 from 450 (Quince et al., 2009). Mothur was also used to cluster sequences into operational taxonomic units (OTUs) and for phylogenetic classification. A 0.03 cutoff (97% identity) was chosen for OTU determination. To visualize the influence of acetylene on OTU abundance, proportional abundances for each OTU were compared for samples collected in the presence vs. absence of acetylene. Outputs were visualized after clustering (using the Euclidean distance method) with Cluster 3.0^2^ using Java TreeView (Saldanha, 2004).

The Primer-E software package (Version 6.0; Clarke and Gorley, 2006) was used to more deeply interrogate relationships between OTUs across samples and to also look for correlations between OTU presence/abundance and environmental parameters. The “shared” file (a matrix file containing OTU abundances for each sample) created by Mothur was imported directly into the Primer-E software package. All OTUs with an abundance (sum of counts across all libraries) >25 sequences were used for further analyses. OTUs meeting these criteria were standardized to the total number of sequences per barcoded library (proportional abundances). These standardized abundances were then square-root transformed to partially deemphasize more highly abundant OTUs. A Bray-Curtis similarity matrix was constructed and used to perform non-metric multidimensional scaling analysis (NMDS) for visualization of community structure relationships between the different samples. The ANOSIM and PERMANOVA programs within Primer-E were used to document the statistical significance of our findings. Both tests were performed on our Bray-Curtis similarity matrix of OTU abundances. A principal components analysis (PCA) was performed on the square-root transformed data to visualize community structure differences and identify OTUs driving the differences in the bacterial communities. Phylogenetic identities of OTUs were determined using the Ribosomal Database Project (RDP) classification within Mothur. We also employed SIMPER to define discriminating species between treatment and control (Clarke, 1993) as well as the software package LEfSe (Segata et al., 2011) to statistically compare differentially abundant OTUs.

## Results

### Net N_2_ Fluxes and N-Fixation Estimates

The net N_2_ flux across the sediment water-interface was quantified to determine if the sediments were net denitrifying (positive N_2_-flux dominated) or net nitrogen fixing (negative N_2_-flux dominated). Net N_2_ fluxes across the sediment-water interface for the individuals cores ranged from -10 to 28.6 μmol N_2_-N m^-2^ h^-1^, suggesting that both N-fixation and denitrification were occurring (**Table 1**). N-fixation rates measured with the ARA, similarly, revealed high variability among the cores and, in some cases rates differed by depth (**Table 1**). After the addition of molybdate, N-fixation rates were reduced on average by almost 70%, indicating that sulfate reducers were responsible for a significant component of the observed N-fixation (Gandy and Yoch, 1988).

**Table 1:** Geochemical measurements of the sediments used in this study.

Core ID	Net N_2_–N flux, μmol m^-2^ h^-1^	Depth, cm	Acetylene reduction assay (ARA)	Treated with Molybdate (M) or Seawater (S)	ARA N_2_ Flux, μmol m^-2^ h^-1^	% change between SW and Mo
Core 1	7.5	0–2	Y	S	22.7	-77
		0–2	Y	M	5.1	
		2–4	Y	S	19.6	-55
		2–4	Y	M	8.9	
		0–2	N	S	n.a.	
		0–2	N	M	n.a.	
		2–4	N	S	n.a.	
		2–4	N	M	n.a.	
Core 2	28.6	0–2	Y	S	27.1	-57
		0–2	Y	M	11.6	
		2–4	Y	S	30.5	-67
		2–4	Y	M	10.0	
		0–2	N	S	n.a.	
		0–2	N	M	n.a.	
		2–4	N	S	n.a.	
		2–4	N	M	n.a.	
Core 3	-10.3	0–2	Y	S	44.3	-60
		0–2	Y	M	17.6	
		2–4	Y	S	62.4	-100
		2–4	Y	M	0.0	
		0–2	N	S	n.a.	
		0–2	N	M	n.a.	
		2–4	N	S	n.a.	
		2–4	N	M	n.a.	

Whole core net N_2_–N fluxes were measured by the N_2_/Ar technique for three cores at the onset of this experiment. These cores were then sub-cored in duplicate, separated by depth (0–2 cm or 2–4 cm), and divided into two groups: a control group not measured by the acetylene reduction assay (ARA) and a treatment group measured with the ARA. In each of these groups, the sub-cores were further divided into two groups: molybdate and seawater treated or seawater treated only. In the sub-cores measured by the ARA we used the commonly employed 3:1 ratio to convert ethylene production to N fixation rate. When geochemical measurements were finished all 24 samples were processed for molecular analysis. (n.a. = not applicable).

### Activity of the Microbial Community

After screening sequences to ensure sufficient quality and length as well as removal of chimeric sequences, 115,319 16S rRNA gene sequences remained across the 24 different samples. Total sequence abundances (per library) ranged from a minimum of 1,379 sequences to a maximum of 10,569 sequences, with an average of 4,805 sequences per library. After clustering, 9,979 OTUs at 0.03 cut-off (97% similarity) remained.

**FIGURE 1:**
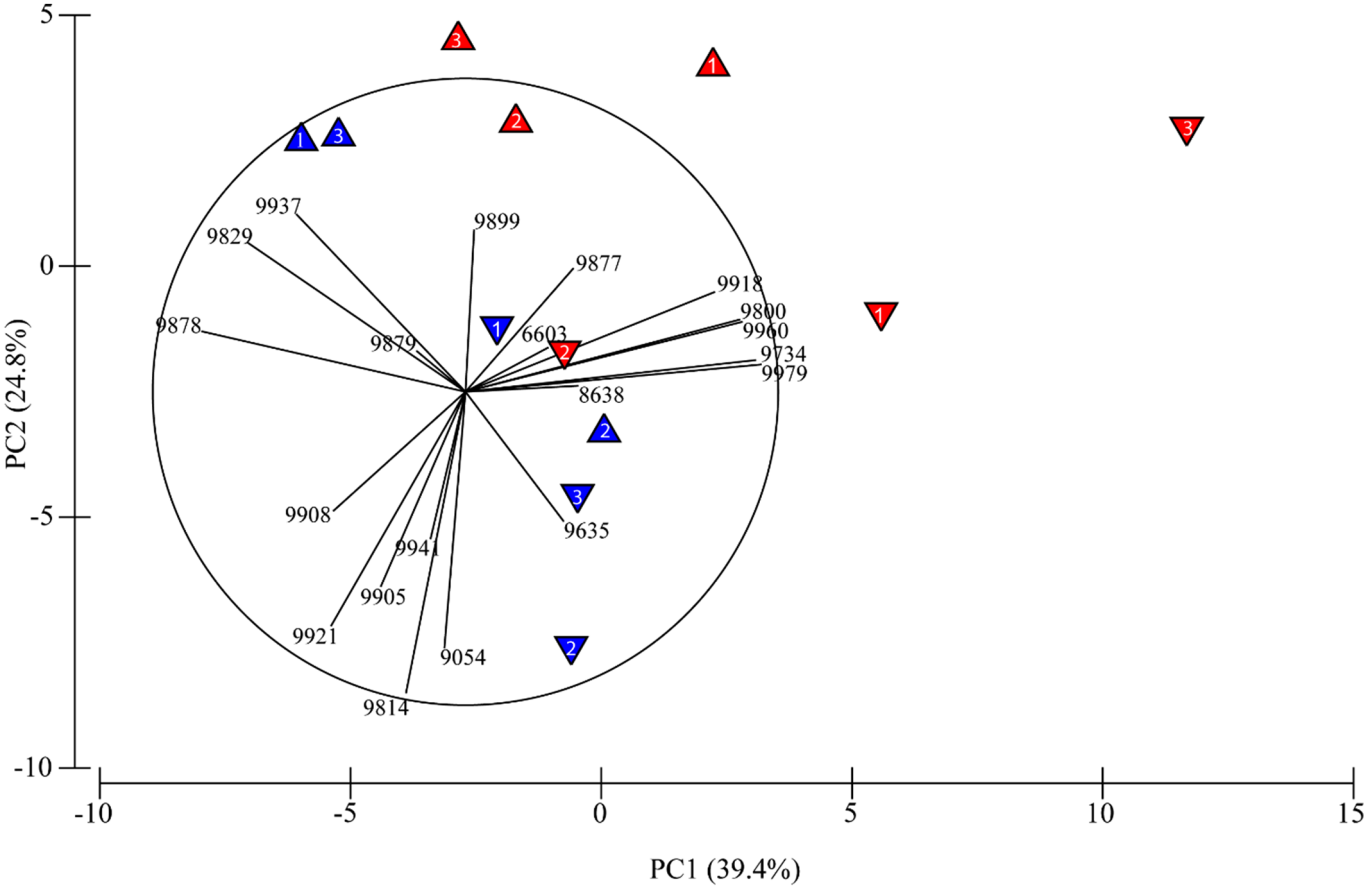
**Results of principal components analysis (PCA) of operational taxonomic units (OTUs) and how they separate between acetylene treated (red triangles) and untreated (blue triangles) and by depth (0–2 cm: upward facing triangles and 2–4 cm: downward facing triangles).** The number in each triangle indicates which core the sample came from. No molybdate treated samples are included here, as molybdate did not significantly alter the active sediment microbial community. Principal component (PC1 and PC2) together, account for 64.2% of the variance in these data.

To determine how acetylene influenced total community structure, we compared total community 16S rRNA expression by principle component analyses (**Figure 1**; **Table 2**). Importantly, we performed PCA analysis only for those samples treated with acetylene and the control groups. Samples treated with molybdate (with or without acetylene) were excluded, as we found no impact of molybdate on the active microbial community (**Table 3**). The PCA highlights two clear patterns. First, there is distinct separation between those samples treated with acetylene and the control along principal component one (PC1). And secondly, depth appears to also be a key driver separating samples along principal component two (PC2). Similarly, the ANOSIM and PERMANOVA analyses show the significant impact of acetylene and depth with the latter being the most significant driver (**Table 3**).

**Table 2:** Dominant operational taxonomic units (OTUs) that drive the separation of the acetylene treated sediments versus the control sediments.

OTU	OTU count	Phylum	Genus (best hit)
**Over-represented in acetylene treatment**
9877^∗^	3738	Proteobacteria	*Thioprofundum* sp.
9979^∗†‡^	3313	Proteobacteria	*Thiohalomonas* sp.
9899^∗†^	1660	Bacteroidetes	Unclassified
9800^∗†‡^	1489	Proteobacteria	*Pelobacter* sp.
9635^∗†^	1044	Bacteroidetes	*Prolixibacter* sp.
9960^∗†‡^	1033	Proteobacteria	*Desulfobulbus* sp.
9734^∗†^	1005	Proteobacteria	*Thiohalomonas* sp.
9918^∗†‡^	912	Bacteroidetes	*Arcicella* sp.
9879^∗^	497	Spirochaetes	*Spirochaeta* sp.
8638^∗†‡^	128	Proteobacteria	*Desulfobacula* sp.
9780^†‡^	165	Proteobacteria	*Pelobacter* sp.
9819^†‡^	98	Proteobacteria	*Thioprofundum* sp.
**Over-represented in control treatment**
9878^∗†‡^	4110	Proteobacteria	*Methylomicrobium* sp.
9814^∗†^	3642	Proteobacteria	*Desulfosalsimonas* sp.
9829^∗†^	2373	Cyanobacteria	*Bacillariophyta* sp.
9905^∗‡^	2328	Bacteroidetes	*Meniscus* sp.
9921^∗†‡^	1912	Bacteroidetes	*Prolixibacter* sp.
9941^∗†^	1436	Bacteroidetes	*Fulvivirga* sp.
9937^∗†^	1347	Bacteroidetes	*Roseivirga* sp.
9054^∗†^	607	Bacteroidetes	*Owenweeksia* sp.
9908^∗†‡^	560	Proteobacteria	*Desulfocapsa* sp.
6603^∗†^	508	Proteobacteria	*Neptuniibacter* sp.
9633^†‡^	516	Bacteroidetes	*Persicobacter* sp.
9608^†‡^	265	Proteobacteria	*Sulfurovum* sp.
5645^†‡^	160	Proteobacteria	*Sedimenticola* sp.
7286^‡^	30	Lentisphaerae	*Lentisphaerae* sp.

OTUs that were in the top 20 of significance in the in the principal components analysis (PCA; ^∗^) and LEfSe analyses (^‡^), along with those deemed significant by SIMPER(†) analyses are noted for discriminating species.

**Table 3:** Results from the ANOSIM and PERMANOVA analyses describing the impact acetylene, depth, or molybdate has on the active sediment microbial community.

Treatment	ANOSIM		PERMANOVA	
	Sample statistic (*R*)	Significance level	Pseudo-F	*p*-value
ARA	0.15	1.1	2.461	0.003
Depth	0.347	0.1	3.739	0.001
Molybdate	-0.016	57.8	0.974	0.495

Together, the first and second components of the PCA explained just over 64% of the variation in the samples (**Figure 1**). The treatments separated primarily along the first component which showed a positive relationship with OTU 9979 (*Thiohalomonas*) and a negative relationship with OTU 9878 (*Methylomicrobium*). The samples also appear to separate by depth along PC2 which is dominated by a negative relationship with OTU 9814 (*Desulfosalsimonas*) and a positive relationship with OTU 9829 (*Bacillariophyta*). In some cases, treatment with acetylene led to increased representation of a number of OTUs within the active community (**Table 2**). Concomitantly, we also observed a number of OTUs that appear to be underrepresented in the acetylene treated versus control groups suggesting that the acetylene may inhibit or alter their activity (**Table 2**). Overall, nine OTUs were identified by all three approaches (PCA, SIMPER, and LEfSe) as significant drivers of difference in the communities while other combinations of approaches (different by SIMPER and LEfSe, 4; different by SIMPER and PCA 9; different by LEfSe and PCA, 1) revealed another 14 OTUs of interest.

**FIGURE 2:**
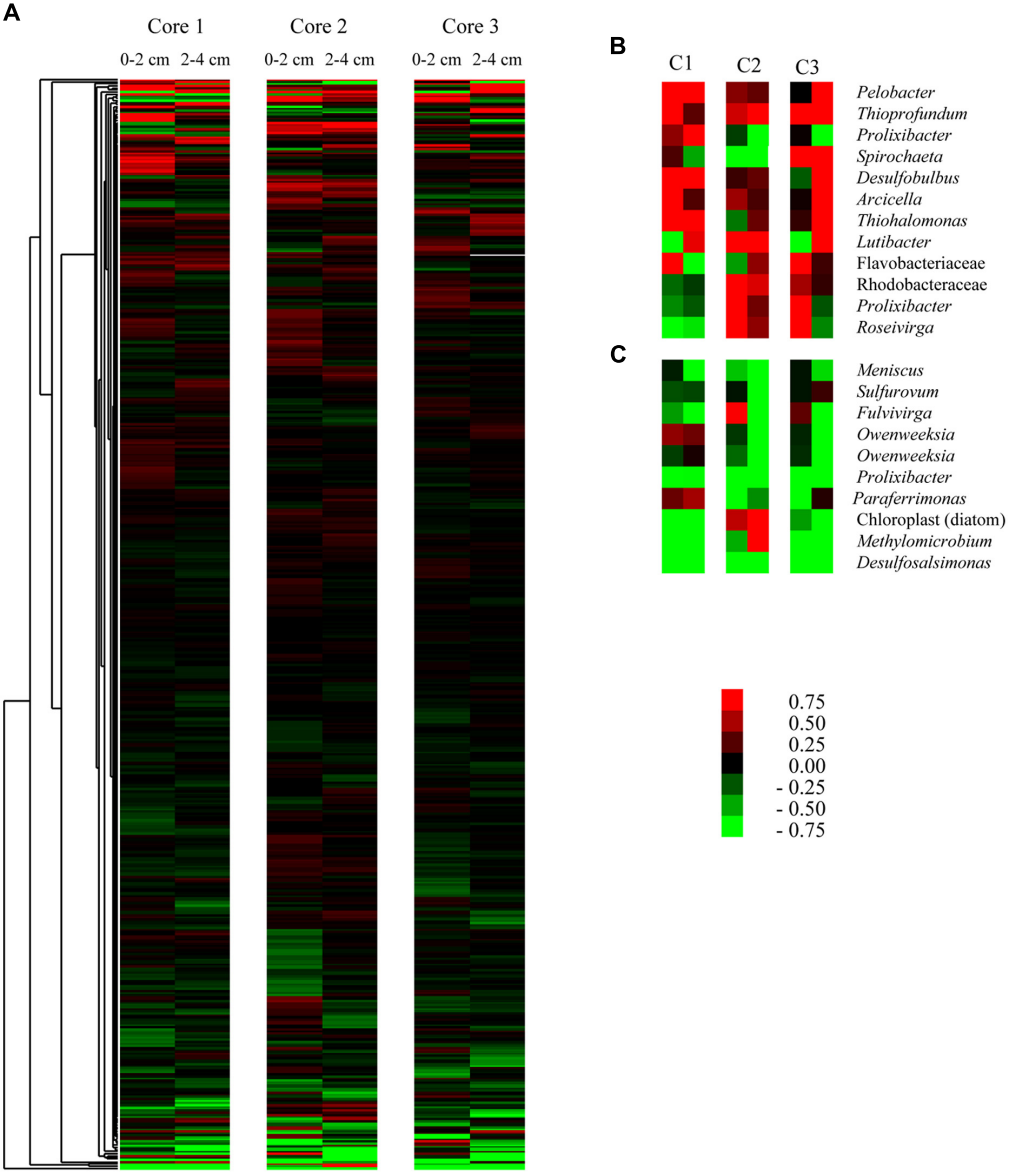
**Influence of acetylene on the distribution of individual (OTUs) from the sediment cores used in this study.** Heat map **(A)** of all OTUs with greater than 25 representative signatures in the complete data set. Data within libraries were normalized to percent of total reads and then expressed as differences between treatment and control [values above zero (red) signify over-representation in the treatment where values below zero (green) represent underrepresentation in the treatment]. Data are shown for three replicate cores and for 0–2 cm and 2–4 cm depth fractions and for seawater treated only (no molybdate). Insets show the 10 most over-represented **(B)** and underrepresented **(C)** groups with cores (C1–C3) and depths (0–2 or 2–4 cm depth) presented in the same order as in **(A)**.

To visualize which OTUs were driving observed relationships, we clustered OTU expression based on differences between control and acetylene-treated replicates (**Figure 2**). We then identified the dominant OTUs that were over-represented under acetylene (**Figure 2B**) and those that were under-represented (**Figure 2C**). Within these groups, changes were subtle: the most upregulated groups (OTUs most closely identified as *Thiohalamonas*, *Thioprofundum,* and *Pelobacter*) demonstrated increases in 16S rRNA representation of up to 9.5%. In parallel, the most down-regulated OTUs in any sample showed decreases in representation of ∼5.0% (*Paraferrimonas* and *Desulfosalimonas*).

## Discussion

### Geochemistry of the Samples

We directly quantified the flux of N_2_ gas across the sediment-water interface using the N_2_/Ar technique. We chose this technique because it is the least intrusive and yet direct measurement of N_2_ flux. Unfortunately, it only provides a net measurement and thus only reports the balance between sediment N-fixation and its opposite process, denitrification (the microbial conversion of nitrate to N_2_). At the time of this experiment, the sediments exhibited net positive N_2_ fluxes (or net denitrification) as well as net negative N_2_ fluxes (or N-fixation; **Table 1**). It is not unusual for sediments at this site to show such variability. In fact, the sediments at this site routinely alternate between net denitrification and net N-fixation and the month following these measurements the sediments were dominated by net N-fixation (Fulweiler and Heiss, 2014). Additionally, we did observe the conversion of acetylene to ethylene for each core and at every depth. The conversion of acetylene to ethylene was considerably reduced when exposed to molybdate suggesting sulfate-reducing bacteria are driving the N-fixation in these sediments. These results are consistent with previous research at this site where N-fixation was observed in core incubations and in large experimental mesocosm studies and, in all cases, the active N-fixing community was dominated by anaerobic sulfate reducers and sulfur/iron reducers (Brown, 2013; Fulweiler et al., 2013; Brown and Jenkins, 2014).

### ARA Impacts on Sulfate Reducing Bacteria and N-Fixation

Several dominant OTUs related to sulfur (S) cycling were observed in this study (**Table 2**). Specifically, we observed OTUs closely related to both sulfur and sulfate reducers (e.g., *Desulfosalsimonas, Desulfobulbus,* etc.) and those related to sulfur oxidizers (e.g., *Thioprofundum and Thiohalomonas*). Sulfur and sulfate reducing bacteria, specifically, *Desulfovibrio* spp. *and Desulfobacter* sp. have long been known to fix N in culture (Sisler and ZoBell, 1951; Widdel, 1987). A rich literature in sea grass beds (Welsh et al., 1996; McGlathery et al., 1998) and salt marshes (Gandy and Yoch, 1988; Piceno and Lovell, 2000) have credited sulfate reducers as the primary N-fixers. More recently, in Baltic Sea sediments, acetylene reduction, and sulfate reduction rates showed similar seasonal patterns and molecular analysis revealed the presence of two sulfate reducing bacteria, *Desulfovibrio vulgaris,* and *Desulfonema limicola* (Bertics and Ziebis, 2010). As the work that motivated this research highlighted, over three decades ago Payne and Grant (1982) reported that acetylene partially or completely inhibited the growth of two sulfate reducing bacteria, *Desulfovibrio desulfuricans,* and *Desulfovibrio gigas* while a third species, *Desulfotomaculum ruminis*, was completely unaffected. In this study, we find a similar species or closely related groups of bacteria responding in different ways to the acetylene (**Figure 2**). The different responses may have important consequences for our understanding of heterotrophic N cycling in marine sediments. Sulfur- and sulfate-reducers are important N-fixers in a range of marine environments, and they experience up or down regulation in the presence of acetylene, one of the most commonly used techniques to measure N-fixation. Thus, we have likely been under- or over-estimating N-fixation rates in these environments. Complicating the picture further is that response appears to be species specific, thus we cannot apply a correction across measurements leaving us with a methodologically introduced unknown amount of error.

These findings are significant because ARA has been and is still widely used in both terrestrial and aquatic ecosystem studies of N-fixation. This may be particularly important for marine systems, where debate exists concerning whether the modern N budget is balanced. Although some estimates have suggested that the oceanic fixed N budget is balanced, they are plagued with gross uncertainties (Gruber, 2004). More recent estimates indicate that the budget is unbalanced with a substantial N deficit, although these budgets also contain considerable uncertainty (Codispoti et al., 1992; Brandes and Devol, 2002; Deutsch et al., 2007). This deficit is driven mainly by larger denitrification rates, as denitrification is thought to be a dominant process in the N cycle, while sediment N fixation is traditionally considered to be inconsequential (Howarth et al., 1988). Thus, to balance the modern marine N budget, rates of denitrification need to be decreased and/or rates of N-fixation increased. Several recent lines of evidence argue for the latter as it appears N-fixation may be more important than originally anticipated (open ocean: Zehr et al., 2001; Davis and McGillicuddy, 2006; shallow coastal systems: Gardner et al., 2006; Fulweiler et al., 2007; Mortazavi et al., 2012). Importantly, these more recent studies have used other techniques besides ARA to measure rates of N-fixation (e.g., N_2_/Ar technique, *nifH* expression, etc.). We propose one important driver delaying our thorough understanding of the role of N-fixation in marine environments is the widespread historic and current use of ARA. Furthermore, because ARA is also used in terrestrial and freshwater ecosystems rates of N-fixation rates may also be inaccurate by varying degrees across these environments.

### ARA Impacts on the Active Microbial Community

We expected to see significant changes in the active microbial community and this was observed in our 16S rRNA bacterial community survey data. We found a significant difference in sediments treated with acetylene versus those in the control samples (**Table 3**). However, the impact of acetylene was not uniform across phylum or genera (**Table 2**). Instead, we observed the up or down regulation of different OTUs associated with Proteobacteria, Spirochaetes, and Bacteroidetes. OTUs dominant under the acetylene treatment most closely identified with a range of bacteria, including *Pelobacter* and *Arcicella*. In contrast, other OTUs were more active in the control samples such as *Methylomicrobium*, *Roseiviriga*, and *Bacillariophyta*.

The data also highlight the heterogenic nature of the sediment microbial community and provide hints about how this heterogeneity alters the community response to disturbance. In this experiment, sediment cores were hand collected by divers within close distance to each other. Despite the careful nature of the collection and the proximity of the sediments, sediment core 2 appears dramatically different to sediments cores 1 and 3 (**Table 1**; **Figure 2**). In core 2 we measured the highest rate of net denitrification (29 μmol N_2_-N m^-2^ h^-1^) and a different microbial response to acetylene as compared to the other cores. The difference in active community response is seen most clearly in the PCA where the 2–4 cm depth sample in core 2 did not change across PC 1. In addition, depth was the most significant driver of differences in the microbial community (**Table 3**). This makes sense as the sediment microbial community was exposed to acetylene for only 7 h and presumably they had much more time to acclimate to there location within the sediments.

Our observations are not surprising as the ARA technique has long been known to inhibit various organisms (Oremland and Taylor, 1975; Schink, 1985; Knowles, 1990). In fact, it is this inhibitory power that has been harnessed to measure denitrification rates in the acetylene block technique (Joye et al., 1996; Welsh et al., 2001) and the importance of methane production and consumption processes (Prior and Dalton, 1985). Thus, as expected we observed repression in the activity of an OTU identifying as *Methylomicrobium*, a strictly aerobic group of methanotrophs (Hanson and Hanson, 1996; Vuilleumier et al., 2012). However, to our knowledge this is the first study to show total active community changes driven by acetylene using 16S rRNA data. These data also allowed us to observe a rapid change in active community metabolism as incubations only lasted 7 h. Overall, our observations concerning the rapid effects of acetylene on microbial community activity give pause concerning the robustness of the technique, adding yet another cautionary note when interpreting ARA data.

## Conflict of Interest Statement

The authors declare that the research was conducted in the absence of any commercial or financial relationships that could be construed as a potential conflict of interest.

## Footnotes

1 http://www.mothur.org/wiki/Schloss_SOP

2 http://bonsai.hgc.jp/~mdehoon/software/cluster/software.htm#ctv
